# The potential effects of climate change on amphibian distribution, range fragmentation and turnover in China

**DOI:** 10.7717/peerj.2185

**Published:** 2016-07-28

**Authors:** Ren-Yan Duan, Xiao-Quan Kong, Min-Yi Huang, Sara Varela, Xiang Ji

**Affiliations:** 1 Jiangsu Key Laboratory for Biodiversity and Biotechnology, College of Life Sciences, Nanjing Normal University, Nanjing, Jiangsu, China; 2 College of Life Sciences, Anqing Normal University, Anqing, Anhui, China; 3 Departamento de Ciencias de la Vida, Edificio de Ciencias, Campus Externo, Universidad de Alcalá, Madrid, Spain; 4 Museum für Naturkunde, Leibniz Institute for Evolution and Biodiversity Science, Berlin, Germany

**Keywords:** MaxEnt, Dispersal, Turnover, Distribution, Climate impacts, Fragmentation, Range shifts, Amphibians

## Abstract

Many studies predict that climate change will cause species movement and turnover, but few have considered the effect of climate change on range fragmentation for current species and/or populations. We used MaxEnt to predict suitable habitat, fragmentation and turnover for 134 amphibian species in China under 40 future climate change scenarios spanning four pathways (RCP2.6, RCP4.5, RCP6 and RCP8.5) and two time periods (the 2050s and 2070s). Our results show that climate change may cause a major shift in spatial patterns of amphibian diversity. Amphibians in China would lose 20% of their original ranges on average; the distribution outside current ranges would increase by 15%. Suitable habitats for over 90% of species will be located in the north of their current range, for over 95% of species in higher altitudes (from currently 137–4,124 m to 286–4,396 m in the 2050s or 314–4,448 m in the 2070s), and for over 75% of species in the west of their current range. Also, our results predict two different general responses to the climate change: some species contract their ranges while moving westwards, southwards and to higher altitudes, while others expand their ranges. Finally, our analyses indicate that range dynamics and fragmentation are related, which means that the effects of climate change on Chinese amphibians might be two-folded.

## Introduction

Global climate is changing rapidly because of anthropogenic greenhouse gas emissions, with unexpected consequences (Solomon, 2007). For example, Solomon (2007) predicted that, if nothing is done to limit the production of greenhouse gases, the average temperature on the earth’s surface is projected to rise by 1.1–6.4 °C between 1990 and 2100. Climate change can alter the distribution of organisms by causing shifts in area, latitude, longitude and/or altitude and thus impact their geographic ranges (Pearson & Dawson, 2003; Raxworthy et al., 2008). Range changes can impact ecosystem function and biodiversity (Raxworthy et al., 2008).

The prediction of climate-driven shifts in species’ potential ranges under future climate scenarios relies on the application of species distribution models (SDMs) (Collevatti et al., 2013; Eskildsen et al., 2013). SDMs use current climate data to model species’ existing distributions, and forecast potential future distributions under various climate scenarios, assuming that species can follow future climate envelopes (Elith & Leathwick, 2009). These models are needed to understand the possible responses of species to future climate change and how current species’ ranges are determined by potential causal factors (Zhang et al., 2012). For example, Pounds et al. (2006) observed a decline in amphibian populations under climate warming using SDMs and Lawler et al. (2006) used SDMs to assess the relative vulnerability of amphibians to future climate change, observing that several regions in Central America will experience high species turnover. More recently, Ochoa-Ochoa et al. (2012) showed that species with a low dispersal capability have high extinction rates, and that climate-driven population declines may be species- and region-specific.

Amphibians are sensitive to changes in thermal and hydric environments due to their unshelled eggs, highly permeable skin and unique biphasic life-cycles (Ochoa-Ochoa et al., 2012; Stuart et al., 2004). At least one third of some 6,000 currently known amphibian species are threatened with extinction, making amphibians one of the most threatened groups of animals on earth (Hof et al., 2011; Stuart et al., 2004). The reasons for this global amphibian decline and rise in threatened species are numerous and complex, but for many species climate change cannot be precluded as a main cause (Stuart et al., 2004; Ficetola et al., 2015).

Locations and regions with many endemic or endangered species, known as hotspots, are more sensitive to climate change (Malcolm et al., 2006). China is a confluence of two main biogeographical divisions, the Oriental and Palaearctic Realms, and contains many priority ecoregions for global conservation (Fei et al., 2009). Some 440 amphibian species, of which 263 are endemic, have been found in China, and the potential remains for further discoveries or redescriptions (http://www.amphibiachina.org). The IUCN (2015) reported that 28% of amphibians in mainland China are threatened or at risk of extinction and 65% of these are endemic. Most of these species are distributed in forests, farmlands and wetlands. Climate change has been hypothesized to have severe synergistic effects on Chinese amphibians because it may exacerbate the adverse effects of habitat destruction and fragmentation associated with anthropogenic land-use change that may increase amphibian extinction risk (Hof et al., 2011). Quantifying the general trends of the climate change-driven shifts in species distribution and abundance is extremely important for adequate conservation policies. However, despite the high endemism and richness of amphibian species in China, to our knowledge, no one has attempted to predict climate change-driven shifts in distribution and abundance.

Many studies showed that climate change causes species’ movement (Pearson & Dawson, 2003; Raxworthy et al., 2008) and significant species turnover (Peterson et al., 2002), but few considered the effect of climate change on fragmentation of current species populations. Here we used MaxEnt (a common SDM) and 40 climate scenarios to study the effect of different greenhouse gas scenarios on the distribution of amphibians in China, assuming that species have no limits to dispersal such that its future distribution becomes the entire area projected by the species distribution model. Our aims are: (1) to quantify the effect of current global warming on Chinese amphibians via potential range shifts, the directions of predicted range shifts and fragmentation of future predicted distributions; (2) calculate the temporal turnover of species composition; and (3) identify priority areas for amphibian conservation across China.

## Materials and Methods

### Species data

Occurrence points for amphibians were collected from the Global Biodiversity Information Facility (GBIF; http://www.gbif.org) and published papers. In order to improve the accuracy of prediction, we did not include species with less than ten different geo-referenced occurrences. We obtained a total of 134 species (20 urodeles of the families Cryptobranchidae (1), Hynobiidae (7) and Salamandridae (12), and 114 anurans of the families Bombinatoridae (3), Bufonidae (6), Dicroglossidae (17), Hylidae (6), Megophryidae (27), Microhylidae (10), Ranidae (35) and Rhacophoridae (10) (Table S1)).

### Climate variables

To build SDMs we chose five climatic variables: (1) annual precipitation; (2) annual mean temperature; (3) temperature seasonality; (4) minimum temperature of the coldest month; and (5) maximum temperature of the warmest month. Although more bioclimatic variables were available we used these five variables because (1) precipitation and temperature are critical climatic factors in all atmospheric ocean general circulation models (AOGCMs) and reflect the availability of water and energy and directly impact amphibian physiology (Collevatti et al., 2013); (2) these variables are very important in determining the distribution of amphibians (Collevatti et al., 2013; Munguía et al., 2012); and (3) the addition of other climatic variables to SDMs generally increases the danger of over-fitting (Collevatti et al., 2013) and the uncertainty (Varela, Lima-Ribeiro & Terribile, 2015).

### Climate layers

Our prediction is based on bioclimatic envelope modeling, which changes with coupled AOGCMs. Different AOGCMs and greenhouse gas scenarios will lead to various changes in species’ distributions in the future. The Intergovernmental Panel on Climate Change (IPCC) in its Fifth Assessment Report (AR5) proposes four Representative Concentration Pathways (RCPs). RCPs may be better than the emission scenarios developed in the Special Report on Emissions Scenarios (SRES) and hence RCPs have replaced SRES standards (Wayne, 2013). The four pathways (RCP2.6, RCP4.5, RCP6 and RCP8.5) represent the four possible radiative forcing values (+2.6, +4.5, +6.0 and +8.5 W/m^2^, respectively) (Wayne, 2013). All climate data were obtained at a 5 arc-min grid scale from WorldClim (http://www.worldclim.org/), and those from 1950–2000 were used as a baseline. Five AOGCMs (Integrated Earth System Model (MIROC-ESM), Beijing Climate Center Climate System Model (BCC-CSM1-1), Goddard Institute for Space Studies (GISS-E2-R), Community Climate System Model (CCSM4) and Institute Pierre Simon Laplace (IPSL-CM5A-LR)) were used for the years 2050s and 2070s. For each AOGCM, we used all four RCPs to evaluate different greenhouse gas scenarios. Hence, the total number of climate scenarios considered was 40 (20 scenarios and two time steps).

### Species distribution modelling

MaxEnt is a commonly used algorithm in species distribution modelling because of its good predictive performance (Elith et al., 2011; Varela et al., 2014). MaxEnt predicts species’ probability distributions of habitat suitability by calculating the maximum entropy distribution and constraining the expected value of each of a set of environmental variables to match the empirical average (Phillips, Anderson & Schapire, 2006). Using presence-only data, MaxEnt fits an unknown probability distribution within the environmental space defined by the input variables of the cells with known species occurrence records. This unknown probability distribution is proportional to the probability of occurrence (Elith et al., 2011).

Analyses were performed in R using the dismo package to simulate species distributions (R Development Core Team, 2013; Hijmans et al., 2015). We carried out SDMs following Elith et al. (2011). For each species, occurrence points were randomly partitioned into two subsets (calibration and validation, at a ratio of 4:1); this was repeated 100 times, each time choosing different random combinations of occurrence points for the calibration/validation datasets. Next, we calculated model parameters and used them to predict future distributions.

The prediction results of the SDMs were evaluated using the area under the receiver operating characteristic curve (AUC) (Elith et al., 2011; Eskildsen et al., 2013; Freeman & Moisen, 2008; Guisan et al., 2013). We used the maximum value of (sensitivity + specificity) as a threshold, in order to minimize the mean of the error rate for both positive and negative observations (Freeman & Moisen, 2008). This is equivalent to maximizing (sensitivity + specificity − 1), otherwise known as the true skill statistic (TSS) (Freeman & Moisen, 2008).

### Species’ range shift and turnover

We used four indicators to illustrate changes in amphibian distribution under climate change scenarios: (1) area change (*AC*); (2) altitude change; (3) latitude change; and (4) longitude change. Area is the number of grid cells occupied by the species and *AC* is the area of a species’ distribution in the future (*A_f_*) minus its current area (*A_c_*), divided by its current area: *AC* = (*A_f_*−*A_c_*)/*A_c_* × 100%. We then calculated the distribution space loss (*DSL*): *DSL* = (*DS_c_*−*DS_fc_*)/*DS_c_* × 100%, new distribution space (*NDS*): *NDS* = (*DS_f_*–*DS_fc_*)/*DS_f_* × 100%, here *DSL* represents the proportional decrease in original distribution area under climate change; *DS_c_* is the distribution space under current climatic scenarios; *DS_f_* is the distribution space under future climatic scenarios; *DS_fc_* is the overlapped distribution space between future and current climatic scenarios; and *NDS* represents the proportion of new distribution area in future distribution under climate change.

To evaluate overall changes in amphibian diversity and distribution in China we calculated species turnover sum (*TS*) and turnover ratio (*TR*) in each grid cell within the potential geographical range shifts for all species. *TS* was calculated as the total number of newly occurring species (*NC*) and extinct species (*NE*) in a given grid cell: *TS* = *NC* + *NE*. *TR* was calculated as *TS* divided by the sum of current species in each grid cell (*NT*) and *NC*: *TR* = *TS*/(*NT + NC*) × 100% (Peterson et al., 2002). In order to choose some significant areas under future change, we artificially set these significant areas had higher *TR* and *TS*, and their numbers were suitable (5∼10). By comparing their different combinations of thresholds of *TR* and *TS*, we used the one-half of its maximum value as the criterion (with *TR* > 50% and *TS* > 20).

### Fragmentation

We studied the fragmentation of species distributions according to methods for calculating habitat fragmentation. We used SDMTools (VanDerWal et al., 2014) to generate patch information from a raster map. To measure species fragmentation we used the coherence index (Jaeger, 2000). The coherence index (*CI*) is a measure of the probability that two animals placed in different patch areas find each other (Jaeger, 2000). The *CI* is calculated as: $CI = \sum\limits_{i = 1}^n {{{\left({{{{A_i}} \over {{A_t}}}} \right)}^2}} $, where *n* is the number of patches; *A_i_* is the size of *i*-th patch; and *A_t_* is the total area of the species distribution. An increase in the *CI* means distribution fragmentation decreases (Jaeger, 2000). We chose the *CI* as our measure and not conventional fragmentation (Cerezo, Perelman & Robbins, 2010) because of (1) its low sensitivity to very small patches as opposed to mean patch size; (2) the monotony of its reaction to different fragmentation phases; and (3) its ability to distinguish spatial patterns.

## Results

**Figure 1:**
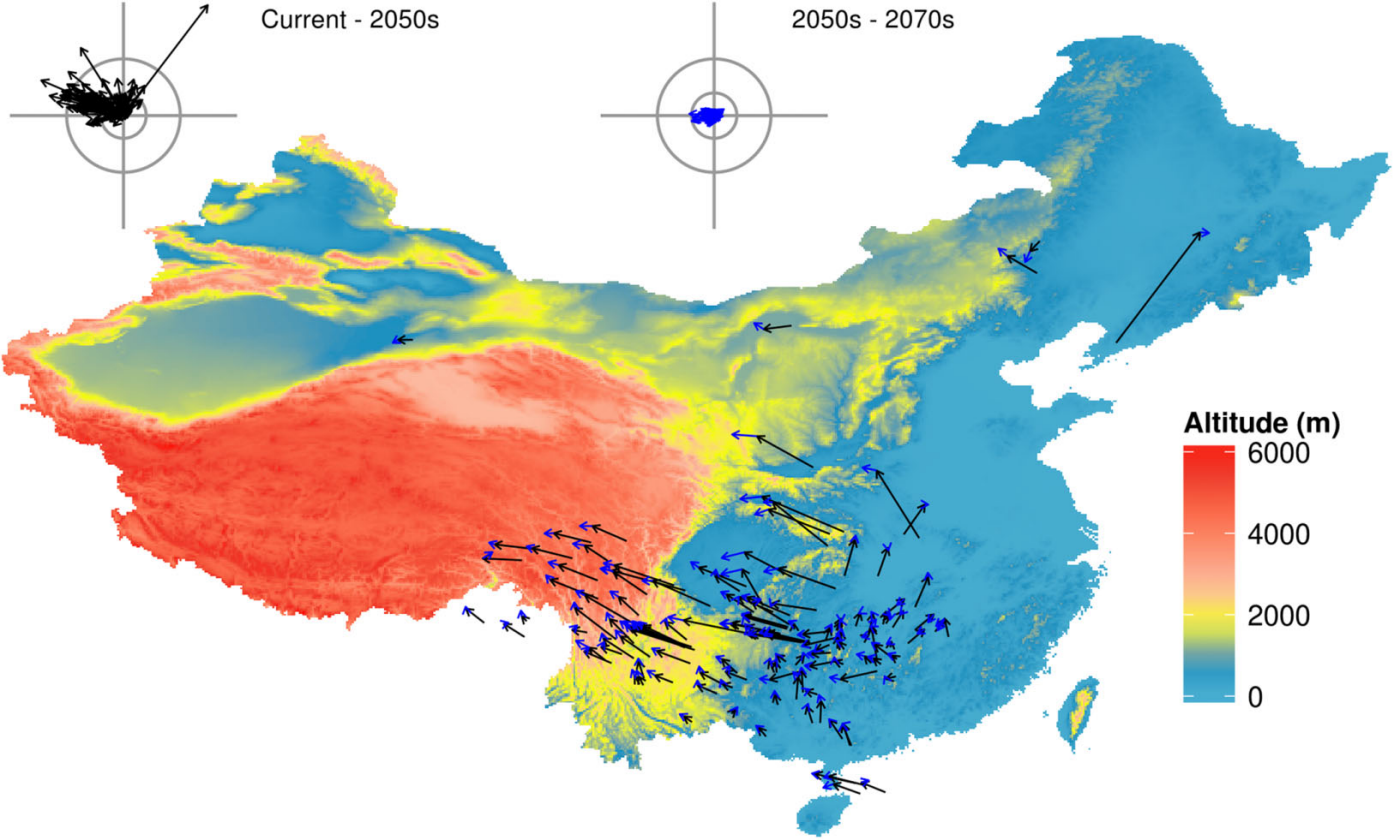
Predicted species movement in a climate scenario, using the BC45 scenario as an example. The arrow represents the distance and direction of species geometric mean point at different periods. The black arrow presents climatic scenario of the 2050s, blue arrow presents climatic scenario of the 2050s–2070s. The wind roses summarize the distance and direction of shift for each species. The radiuses of rings on each wind rose represent geographical distance (inner circus: 2°; outer circus: 5°). The grey axis bars on wind roses represent a length of 7°. BC45 scenario represents BCC-CSM1-1 as AOGCM and using RCP4.5 as greenhouse gas scenarios. The figure was generated using R (http://www.R-project.org/), ggplot2 (https://cran.r-project.org/web/packages/ggplot2/index.html) and raster (http://CRAN.R-project.org/package=raster) softwares, and the map was created using data downloaded from the GADM database (http://www.gadm.org/) for free use.

MaxEnt shows great predictive performance for all distributions under the baseline scenario, with high values for AUC (> 0.8). The 134 amphibians show varying sensitivities to future climate change and most species have large changes in RCP8.5 in the 2070s (Figs. 1, S1 and S2).

**Figure 2:**
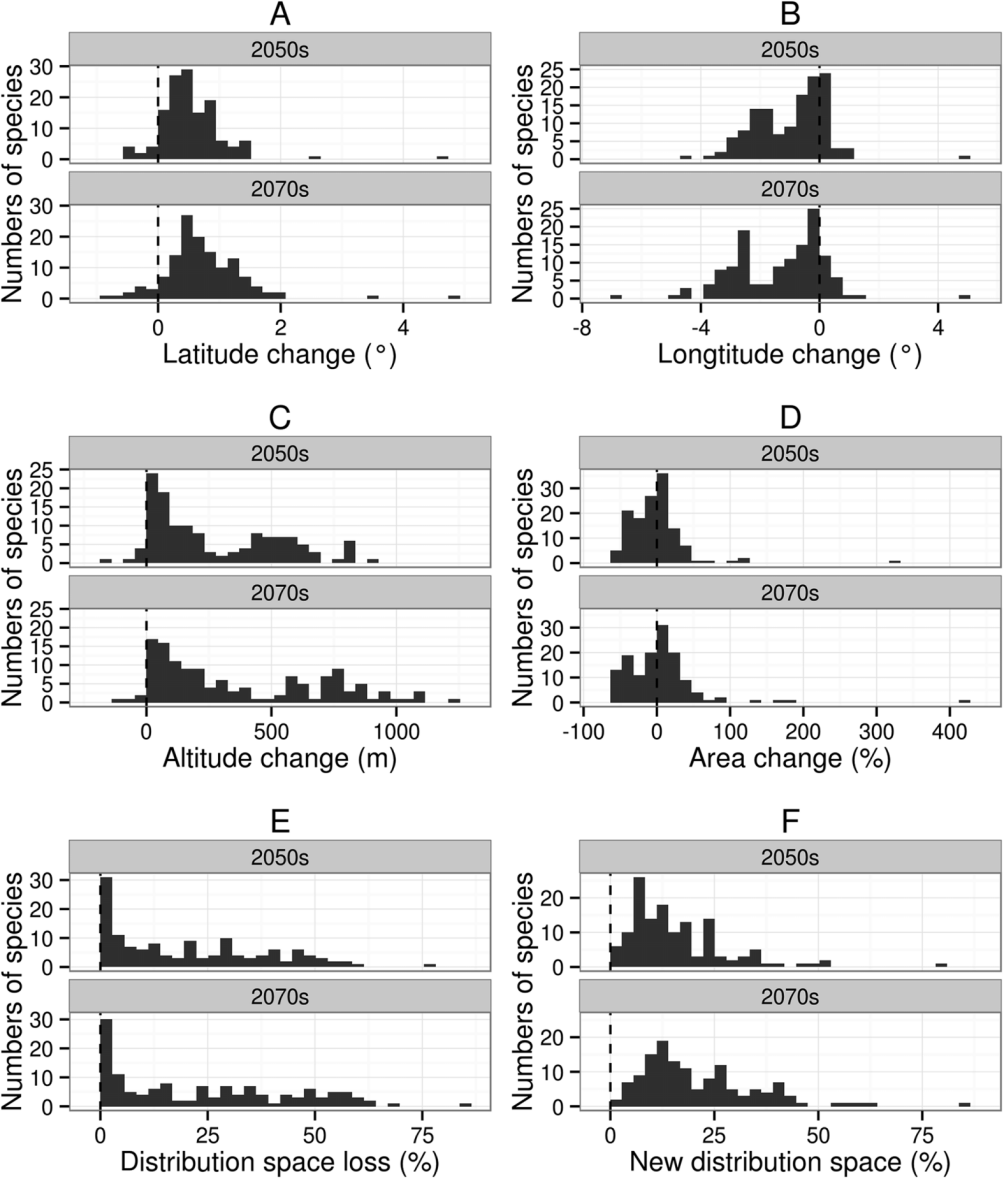
Distribution patterns of 134 species of amphibians from different aspects. Sub-graph (A) present latitude change of 134 kinds of amphibians. (B) present longitude change. (C) present altitude change. (D) present area change percent. (E) present distribution space loss percent. (F) new distribution space percent.

The suitable habitat of the majority of species (92.5% in the 2050s, and 91.8% in the 2070s) will move northwards (mean latitude increased), with a mean latitude shift of 0.60° by the 2050s and 0.83° by the 2070s (Fig. 2A). The suitable habitat of the majority of species (76.9% in the 2050s, and 84.3% in the 2070s) will move westwards (mean longitude will decrease) across all future scenarios ranging from 0.03–4.51° (mean 1.35°) in the 2050s, and from 0.03–6.87° (mean 1.72°) in the 2070s. The number of species with the furthest longitudinal movement (more than 0.5° and more than 1°) are 75 and 56 in the 2050s, respectively, and 84 and 68 in the 2070s (Fig. 2B). Virtually all species (95.5% in the 2050s, and 97.0% in the 2070s) will move to higher altitudes under climate change (from currently 137–4,124 m to 286–4,396 m in the 2050s or 314–4,448 m in the 2070s), with a mean range shift of 287.2 m by the 2050s and 387.8 m by the 2070s (Fig. 2C).

Area change will vary from −52.8–324.5% by the 2050s and from −57.6–418.1% by the 2070s. The 70.9% of species in the 2050s (38.1% for area contraction and 32.8% for area expansion) and 75.4% of species in the 2070s (37.3% for area contraction and 38.1% for area expansion) will undergo a significant change in distribution of greater than 10% (Fig. 2D). Among these species, three and six species in the 2050s, and 13 and 11 species in the 2070s will respectively show substantial area contraction (greater than 50%) and expansion (greater than 50%) (Fig. 2D).

By the 2050s, the mean value of DSL will be 20.7%, and nine species will lose more than 50% of their original distribution space; by the 2070s, the mean value of DSL will be 23.9%, and 22 species will lose more than 50% of their original distribution space (Fig. 2E). By the 2050s, the mean value of the NDS ratio for amphibians will be 15.9%, and three species will have a NDS greater than 50%; by the 2070s the mean value of the NDS ratio will be 21.1%, and five species will have a NDS greater than 50% (Fig. 2F).

Area change and area change ratio were correlated with changes in latitude, longitude and altitude (Table 1). In other words, under climate change, suitable habitat of amphibians that move westwards, southwards and to higher altitudes will undergo overall range contraction.

**Table 1:** Correlation coefficients between parameters.

	2050s		2070s	
	Area change	Area change ratio (%)	Area change	Area change ratio (%)
Current area	0.363***	0.108	0.358***	0.069
Current latitude	0.058	0.135	0.049	0.118
Current longitude	0.053	0.226**	0.060	0.220*
Current altitude	−0.074	−0.146	−0.084	−0.144
Latitude change	0.28**	0.516***	0.355***	0.524***
Longitude change	0.340***	0.477***	0.371***	0.464***
Altitude change	−0.405***	−0.374***	−0.432***	−0.373***
New distribution area	−0.027	−0.116	−0.016	−0.123
Distribution area loss	−0.011	−0.074	−0.012	−0.072
Change of coherence index	0.656***	0.517***	0.624***	0.534***

**Notes:**

* *P* < 0.05.

** *P* < 0.01.

*** *P* < 0.001.

**Figure 3:**
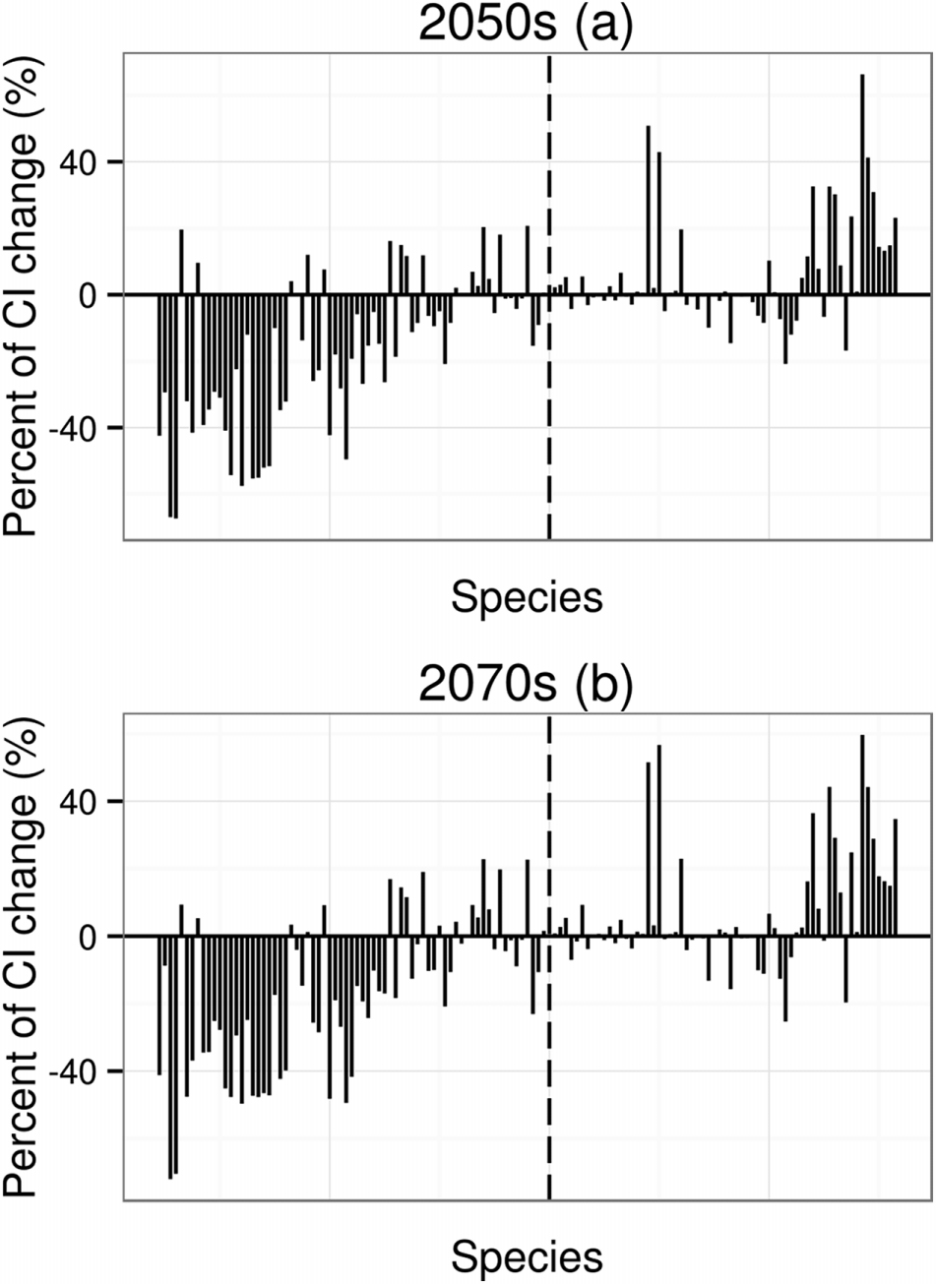
Percent of coherence index (*CI*) change. *CI* is the probability that two animals placed in different areas (patches) will find each other. The order of 134 species in *X* axis from left to right depends on the order of mean value of area change (from low to high, to make thing to be comparable, the 2070s using the order of the 2050s). The dotted line means that the potential distribution area has no any change under future climatic change.

For species undergoing declines in distribution, the mean value of coherent index (*CI*) change will be −16.2% for the 2050s and −19.6% for the 2070s; for species undergoing increases in distribution, the mean value of *CI* change will be 5.9% for the 2050s and 6.6% for the 2070s. Under climate change, species with higher area change (decrease or increase) will have higher *CI* changes (Fig. 3).

**Figure 4:**
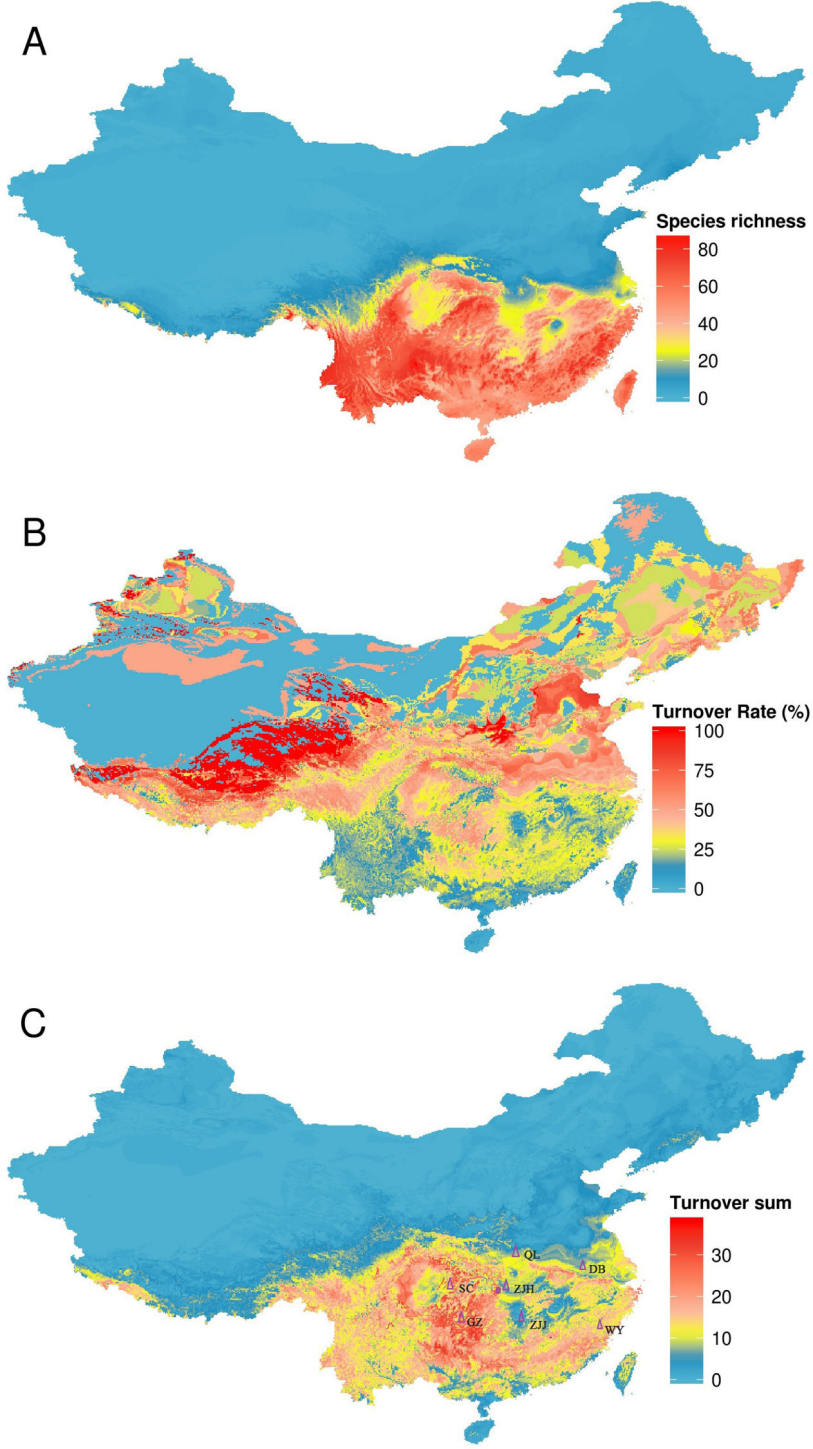
Turnover of species under climate change, using the BC45 scenario in the 2070s as example. (A) species richness in current; (B) turnover rate; (C) turnover sum of 134 species. The figure was generated using R (http://www.R-project.org/), ggplot2 (https://cran.r-project.org/web/packages/ggplot2/index.html) and raster (http://CRAN.R-project.org/package=raster) softwares, and the map was created using data downloaded from the GADM database (http://www.gadm.org/) for free use. QL, Qinling Mountains; DB, Dabie Mountains; SC, Sichuan Basin and surrounding areas; GZ, western Guizhou province; WY, Wuyi Mountains; ZJJ, Zhangjiajie Nature Reserve; ZJH, Zhongjianhe Nature Reserve.

Different regions have different *TR* and *TS* (Fig. 4). Areas with the highest *TR* are located in Northwest China where amphibian species richness is lower. Areas with high *TS* are located in Central and Southern China and these areas were inconsistent with areas of high *TR*. According to our combining indicator (with *TR* > 50% and *TS* > 20) (one-half of its maximum value as the criterion), climate strongly influenced amphibian distributions in five regions: the Qinling Mountains, Wuyi Mountains, Dabie Mountains, Sichuan Basin and surrounding areas, and western Guizhou province (Fig. 4).

## Discussion

Climatic shifts to warmer and drier regimes can have profound effects on the distribution of amphibians (Araújo, Thuiller & Pearson, 2006). In accordance to this, our results show that Chinese amphibians would exhibited a variety of climate-driven range shifts and as expected we detected the greatest change in amphibian distribution under RCP8.5 and by the 2070s.

### Effects of climate change on the direction of movement

The average temperature of the earth’s surface will rise by up to 6.4 °C by 2100, and species would thus need to migrate to higher latitudes and/or elevations following their climatic requirements (Pearson & Dawson, 2003; Raxworthy et al., 2008). When temperature undergoes one degree change, elevation needs to change 100–200 m and latitude about 0.5° (about 55 km of polar movement, though latitude has a complex and variable relationship with temperature) (Peterson & Vose, 1997). Our study confirmed these general trends and that under climate warming the suitable habitat of Chinese amphibians will predominantly migrate to higher altitudes and latitudes, which has the same movement direction reported for amphibians in other regions (Mokhatla, Rödder & Measey, 2015; Cunningham et al., 2016). For example, Araújo, Thuiller & Pearson (2006) suggest that, a great proportion of the 108 herpetofaunal species (42 amphibians and 66 reptiles) in Europe would expand their distribution northward by 2050. Forero-Medina, Joppa & Pimm (2011) project that 46 amphibian species on a tropical mountain will shift to higher elevations under future climate changes, and they further document that higher elevation habitats will be more isolated than previously, where is unlikely to sustain their survival and reproduction. However, the direction and speed of migration depend on the climate scenario and species modelled.

Also, our analysis showed that the majority of amphibians will move westwards. This result contradicts other studies where no trend in longitudinal displacement was found (Peterson et al., 2002). However, the longitudinal trend observed in China is plausible given that the terrain of the country is high in the west and low in the east (amphibians will move to higher altitudes under climate warming), and that East China is adjacent to the sea without space for amphibians to migrate.

Organisms often show species-specific environmental requirements and global climate change has different effects on the ranges of different species. For example, Lawler et al. (2010) predict that amphibian species in several areas in Central and western South America would experience high species turnover and would experience larger range contractions by 2071. Li et al. (2013) assess the vulnerability of 208 endemic or endangered species (including 13 amphibians) in China and predict that climate-induced shifts in ranges will lead 50% of species to undergo range reduction another half to increase range. Foden et al. (2013) document that 11–15% of amphibians, 6–9% of birds and 6–9% of coral species are highly vulnerable to climate change. Our study confirmed that the responses of amphibians to future climate change are complex: some species will undergo distribution reduction, whereas others will expand their ranges. Following our results, amphibians that move westwards (drier habitats), southwards (warmer habitats) and to higher altitudes will undergo distribution reduction. The main reason may be that compared with the original habitats of amphibians, there are fewer suitable habitats in the west and south parts of China where there are many mountains.

### Effects of climate change on fragmentation

Our study shows that under climate warming an increase in fragmentation (lower *CI*) will decrease distribution areas and increase the rate of extinction. Fragmentation can reduce population size and habitat connectivity, interfere with gene interchange, and reduce migration rates and resilience (Chen & Bi, 2007; Sarmento Cabral et al., 2013), thus negatively affecting the long-term viability of threatened and endangered amphibians (Opdam & Wascher, 2004).

Further, our study shows that the lost habitat for some species is not at the edge of distributions but mainly in the core (central) region of their ranges (Fig. S3). The core distribution region is very important for a species because it acts as a hub that connects patches, allowing the genetic exchange between different populations. Habitat loss and fragmentation have been identified as one of the major causes of amphibian decline globally (Stuart et al., 2004). Our study shows that future climate change might not only reduce the range of some amphibians, but also make it more fragmented. This finding is similar to that reported for amphibians in other regions (Mokhatla, Rödder & Measey, 2015). The observed synergic effect would accelerate the decline and/or local extinction of certain amphibians (e.g. *Amolops ricketti*, *Pachytriton labiatus* and *Megophrys minor*). On the other hand, species predicted to undergo area expansion such as *Hynobius leechii*, *Hylarana macrodactyla* and *Fejervarya multistriata* were not affected by fragmentation, which would benefit them and allow them to expand more easily.

### Species turnover and high impact areas

The identification of critical habitats for amphibian protection under climate change is important for making robust conservation management decisions (Guisan et al., 2013). Areas of high species turnover may be sites with largest shifts in population. Many studies conduct turnover assessments using turnover ratios (Erasmus et al., 2002; Peterson et al., 2002), however our results revealed that areas with high turnover ratios were not the same as areas with high turnover sums. This is because an area with a low turnover sum can have a high turnover ratio if the area has a very low species richness under the current climate (e.g. northwestern China). We considered grid cells with turnover ratios greater than 50% and turnover sums greater than 20 as areas of potentially large future shifts in amphibians. We found several such areas including the Sichuan Basin and surrounding areas, the Qinling Mountains, the Dabie Mountains, the Wuyi Mountains and western Guizhou, and hypothesize that these regions may see major shifts in amphibians as a result of the combined action of several factors. First, the Sichuan Basin and surrounding areas, western Guizhou province and Dabie Mountains are located in an area of transition from the northern subtropics to warm temperate climate; there are relatively large climatic gradients in these areas (Xie et al., 2007). Second, these five areas contain the boundaries of many species’ distributions (Fei et al., 2009); areas containing many range limits are expected to experience greater turnover than those containing few range limits. Third, mountainous regions, such as the Qinling Mountains form a natural (north or south) boundary for many species and so may experience significant faunal change. Under climate change, habitat loss, especially that resulting from changes to freshwater ecosystems, is the greatest risk to amphibians (Solomon, 2007).

### Conservation implications

We found overlapping key amphibian regions, such as important endemic amphibian regionalization (e.g. Sichuan and Guizhou provinces) and global biodiversity hotspots (e.g. Sichuan) (Chen & Bi, 2007). Nature reserves provide the most effective approach for biodiversity conservation, especially for the in situ conservation of wildlife and natural ecosystems (D’Amen et al., 2011). Distribution shift, habitat loss and fragmentation caused by climatic change are the potential threats to amphibians in China, and the current network of natural reserves does not include some key regions for amphibians. In addition, many existing nature reserves have not sufficient to prevent amphibians from declining from threats of climatic change because their conservation objects and policies are not for amphibians, though they have a key role in protect amphibians. We should protect the amphibians under the climate change with the following strategies. First, we need to develop a conservation plan to estab1ish a network of national reserves covering populations of all threatened amphibian species in China. Second, we need to implement conservation action plans in all currently existing reserves to maintain viable populations and protect the habits for some vulnerable species with high habit loss (e.g. *Odorrana hainanensis*, *Cynops cyanurus*, *Bombina fortinuptialis*, *Rhacophorus dennysi*, *Rana amurensis*, *Tylototriton asperrimus*, *Pelophylax hubeiensis* and *Bufo melanostictus*) and local extinction of certain amphibians for fragmentation (e.g. *Amolops ricketti*, *Pachytriton labiatus* and *Megophrys minor*). Third, we need to develop some engineering strategies to increase shelters and canopy cover and install irrigation to maintain water potentials in wetland and upland habitats (Li, Cohen & Rohr, 2013).

### Methodological limitations

Our models assume that only climatic variables affect species range, and that all species have the potential to migrate to areas climatically suitable for them. Many important factors such as vegetation canopy, land use, movement barriers, mountain topography and biological characteristics of focal species were not taken into account. Our results therefore simplify the real effects of climate change because amphibians in nature are affected by numerous biotic and abiotic factors (Gotelli, Graves & Rahbek, 2010; Nakazawa, 2013; Trisurat, Kanchanasaka & Kreft, 2015). However, this simple picture does provide useful information on the potential effects of future climate change on Chinese amphibians, and their possible trends of migration. Our study is the first to investigate how Chinese amphibians would respond to future climate change.

## Supplemental Information

10.7717/peerj.2185/supp-1 Supplemental Information 1 Raw data.

10.7717/peerj.2185/supp-2 Supplemental Information 2 Scientific classification and IUCN category of 134 amphibian species analyzed.

10.7717/peerj.2185/supp-3 Supplemental Information 3 Species movement under different AOGCM models and RCP in the 2050s. Y axis presents different AOGCM models. X axis presents different RCP models. The arrow and wind rose are same as Fig. 1.

10.7717/peerj.2185/supp-4 Supplemental Information 4 Species movement under different AOGCM models and RCP in the 2070s. Y axis presents different AOGCM models. X axis presents different RCP models. The arrow and wind rose are same as Fig. 1.

10.7717/peerj.2185/supp-5 Supplemental Information 5 Distribution change under climate change using Megophrys major as an example. The figure was generated using R (http://www.R-project.org/), ggplot2 (http://had.co.nz/ggplot2/boo) and raster (http://CRAN.R-project.org/package=raster) softwares, and the maps were created using data downloaded from the GADM database (http://www.gadm.org/) for free use.

## Additional Information and Declarations

### Data Deposition

The following information was supplied regarding data availability: The raw data has been supplied as Supplemental Dataset Files.
