# Supplementary material for: The potential effects of climate change on amphibian distribution, range fragmentation and turnover in China
Source: PeerJ. 2016 Jul 28;4:e2185. doi: 10.7717/peerj.2185 (PMC4974927; doi:10.7717/peerj.2185)
Supplement: Supplemental Information 2 [file peerj-04-2185-s002.doc]

**Table S1**

Scientific classification and IUCN category of 134 amphibian species analyzed. CR: critically endangered; DD: data deficient; EN: endangered; LC: least concern; NE: not evaluated; NT: near threatened; VU: vulnerable.

| Species | Family | Order | IUCN category |
| --- | --- | --- | --- |
| *Andrias davidianus* | Cryptobranchidae | Caudata | CR |
| *Batrachuperus cochranae* | Hynobiidae | Caudata | VU |
| *Batrachuperus londongensis* | Hynobiidae | Caudata | EN |
| *Batrachuperus pinchonii* | Hynobiidae | Caudata | VU |
| *Batrachuperus tibetanus* | Hynobiidae | Caudata | VU |
| *Batrachuperus yenyuanensis* | Hynobiidae | Caudata | VU |
| *Hynobius chinensis* | Hynobiidae | Caudata | EN |
| *Hynobius leechii* | Hynobiidae | Caudata | LC |
| *Cynops cyanurus* | Salamandridae | Caudata | LC |
| *Cynops orientalis* | Salamandridae | Caudata | LC |
| *Pachytriton brevipes* | Salamandridae | Caudata | LC |
| *Pachytriton labiatus* | Salamandridae | Caudata | LC |
| *Paramesotriton caudopunctatus* | Salamandridae | Caudata | NT |
| *Paramesotriton chinensis* | Salamandridae | Caudata | LC |
| *Tylototriton asperrimus* | Salamandridae | Caudata | NT |
| *Tylototriton kweichowensis* | Salamandridae | Caudata | VU |
| *Tylototriton shanjing* | Salamandridae | Caudata | NT |
| *Tylototriton taliangensis* | [Salamandridae](http://fr.wikipedia.org/wiki/Salamandridae) | Caudata | NT |
| *Tylototriton verrucosus* | [Salamandridae](http://fr.wikipedia.org/wiki/Salamandridae) | Caudata | LC |
| *Tylototriton wenxianensis* | [Salamandridae](http://fr.wikipedia.org/wiki/Salamandridae) | Caudata | VU |
| *Bombina fortinuptialis* | Bombinatoridae | Anura | NE |
| *Bombina maxima* | Bombinatoridae | Anura | NE |
| *Bombina microdeladigitora* | Bombinatoridae | Anura | NE |
| *Bufo gargarizans* | Bufonidae | Anura | LC |
| *Bufo melanostictus* | Bufonidae | Anura | LC |
| *Bufo pewzowi* | Bufonidae | Anura | NE |
| *Bufo tibetanus* | Bufonidae | Anura | LC |
| *Bufo tuberculatus* | Bufonidae | Anura | NT |
| *Strauchbufo raddei* | Bufonidae | Anura | LC |
| *Fejervarya limnocharis* | Dicroglossidae | Anura | LC |
| *Fejervarya multistriata* | Dicroglossidae | Anura | DD |
| *Fejervarya quadranus* | Dicroglossidae | Anura | NE |
| *Hoplobatrachus chinensis* | Dicroglossidae | Anura | NE |
| *Limnonectes fragilis* | Dicroglossidae | Anura | VU |
| *Limnonectes kuhlii* | Dicroglossidae | Anura | LC |
| *Nanorana liui* | Dicroglossidae | Anura | VU |
| *Nanorana parkeri* | Dicroglossidae | Anura | LC |
| *Nanorana pleskei* | Dicroglossidae | Anura | NT |
| *Nanorana yunnanensis* | Dicroglossidae | Anura | EN |
| *Occidozyga lima* | Dicroglossidae | Anura | LC |
| *Quasipaa boulengeri* | Dicroglossidae | Anura | EN |
| *Quasipaa exilispinosa* | Dicroglossidae | Anura | VU |
| *Quasipaa robertingeri* | Dicroglossidae | Anura | EN |
| *Quasipaa shini* | Dicroglossidae | Anura | VU |
| *Gynandropaa sichuanensis* | Dicroglossidae | Anura | NE |
| *Quasipaa spinosa* | Dicroglossidae | Anura | VU |
| *Hyla chinensis* | Hylidae | Anura | LC |
| *Hyla gonshanensis* | Hylidae | Anura | NE |
| *Hyla japonica* | Hylidae | Anura | LC |
| *Hyla sanchiangensis* | Hylidae | Anura | LC |
| *Hyla simplex* | Hylidae | Anura | LC |
| *Hyla tsinlingensis* | Hylidae | Anura | LC |
| *Megophrys boettgeri* | Megophryidae | Anura | DD |
| *Megophrys daweimontis* | Megophryidae | Anura | NE |
| *Megophrys huangshanensis* | Megophryidae | Anura | DD |
| *Megophrys jingdongensis* | Megophryidae | Anura | LC |
| *Megophrys major* | Megophryidae | Anura | LC |
| *Megophrys mangshanensis* | Megophryidae | Anura | NE |
| *Megophrys minor* | Megophryidae | Anura | LC |
| *Megophrys omeimontis* | Megophryidae | Anura | LC |
| *Megophrys wuliangshanensis* | Megophryidae | Anura | NE |
| *Megophrys wushanensis* | Megophryidae | Anura | NE |
| *Leptobrachium boringii* | Megophryidae | Anura | EN |
| *Leptobrachium leishanensis* | Megophryidae | Anura | EN |
| *Leptobrachium jiulongshanensis* | Megophryidae | Anura | NE |
| *Leptolalax oshanensis* | Megophryidae | Anura | LC |
| *Leptolalax pelodytoides* | Megophryidae | Anura | LC |
| *Oreolalax lichuanensis* | Megophryidae | Anura | NT |
| *Oreolalax major* | Megophryidae | Anura | VU |
| *Oreolalax pingii* | Megophryidae | Anura | EN |
| *Oreolalax popei* | Megophryidae | Anura | LC |
| *Oreolalax rhodostigmatus* | Megophryidae | Anura | VU |
| *Oreolalax rugosus* | Megophryidae | Anura | NT |
| *Oreolalax schmidti* | Megophryidae | Anura | NT |
| *Scutiger boulengeri* | Megophryidae | Anura | LC |
| *Scutiger glandulatus* | Megophryidae | Anura | LC |
| *Scutiger mammatus* | Megophryidae | Anura | LC |
| *Scutiger nyingchiensis* | Megophryidae | Anura | LC |
| *Scutiger tuberculatus* | Megophryidae | Anura | VU |
| *Calluella yunnanensis* | Microhylidae | Anura | LC |
| *Kaloula interlineatus* | Microhylidae | Anura | NE |
| *Kaloula pulchra* | Microhylidae | Anura | LC |
| *Microhyla butleri* | Microhylidae | Anura | LC |
| *Microhyla heymonsi* | Microhylidae | Anura | LC |
| *Microhyla mixtura* | Microhylidae | Anura | LC |
| *Microhyla ornata* | Microhylidae | Anura | LC |
| *Microhyla pulchra* | Microhylidae | Anura | LC |
| *Kaloula rugifera* | Microhylidae | Anura | LC |
| *Kaloula verrucosa* | Microhylidae | Anura | LC |
| *Amolops chunganensis* | Ranidae | Anura | LC |
| *Amolops granulosus* | Ranidae | Anura | LC |
| *Amolops hainanensis* | Ranidae | Anura | EN |
| *Amolops lifanensis* | Ranidae | Anura | NT |
| *Amolops loloensis* | Ranidae | Anura | VU |
| *Amolops mantzorum* | Ranidae | Anura | LC |
| *Amolops ricketti* | Ranidae | Anura | LC |
| *Amolops torrentis* | Ranidae | Anura | VU |
| *Amolops viridimaculatus* | Ranidae | Anura | NT |
| *Amolops wuyiensis* | Ranidae | Anura | LC |
| *Hylarana cubitalis* | Ranidae | Anura | LC |
| *Hylarana guentheri* | Ranidae | Anura | LC |
| *Hylarana latouchii* | Ranidae | Anura | LC |
| *Hylarana macrodactyla* | Ranidae | Anura | LC |
| *Hylarana nigrotympanica* | Ranidae | Anura | LC |
| *Hylarana nigrovittata* | Ranidae | Anura | LC |
| *Hylarana spinulosa* | Ranidae | Anura | VU |
| *Hylarana taipehensis* | Ranidae | Anura | LC |
| *Odorrana andersonii* | Ranidae | Anura | LC |
| *Odorrana grahami* | Ranidae | Anura | NT |
| *Odorrana graminea* | Ranidae | Anura | DD |
| *Odorrana hainanensis* | Ranidae | Anura | VU |
| *Odorrana margaretae* | Ranidae | Anura | LC |
| *Odorrana schmackeri* | Ranidae | Anura | LC |
| *Odorrana versabilis* | Ranidae | Anura | LC |
| *Pelophylax fukienensis* | Ranidae | Anura | LC |
| *Pelophylax hubeiensis* | Ranidae | Anura | LC |
| *Pelophylax nigromaculatus* | Ranidae | Anura | NT |
| *Pelophylax plancyi* | Ranidae | Anura | LC |
| *Pseudorana weiningensis* | Ranidae | Anura | VU |
| *Rana adenopleura* | Ranidae | Anura | LC |
| *Rana amurensis* | Ranidae | Anura | LC |
| *Rana chaochiaoensis* | Ranidae | Anura | LC |
| *Rana chensinensis* | Ranidae | Anura | LC |
| *Rana omeimontis* | Ranidae | Anura | LC |
| *Buergeria oxycephala* | Rhacophoridae | Anura | VU |
| *Polypedates megacephalus* | Rhacophoridae | Anura | LC |
| *Rhacophorus chenfui* | Rhacophoridae | Anura | LC |
| *Rhacophorus dennysi* | Rhacophoridae | Anura | LC |
| *Rhacophorus dugritei* | Rhacophoridae | Anura | LC |
| *Rhacophorus gongshanensis* | Rhacophoridae | Anura | NT |
| *Rhacophorus hungfuensis* | Rhacophoridae | Anura | DD |
| *Rhacophorus nigropunctatus* | Rhacophoridae | Anura | NT |
| *Rhacophorus omeimontis* | Rhacophoridae | Anura | LC |
| *Rhacophorus rhodopus* | Rhacophoridae | Anura | LC |
