# Supplementary figures and images for: The potential effects of climate change on amphibian distribution, range fragmentation and turnover in China
Source: PeerJ. 2016 Jul 28;4:e2185. doi: 10.7717/peerj.2185 (PMC4974927; doi:10.7717/peerj.2185)

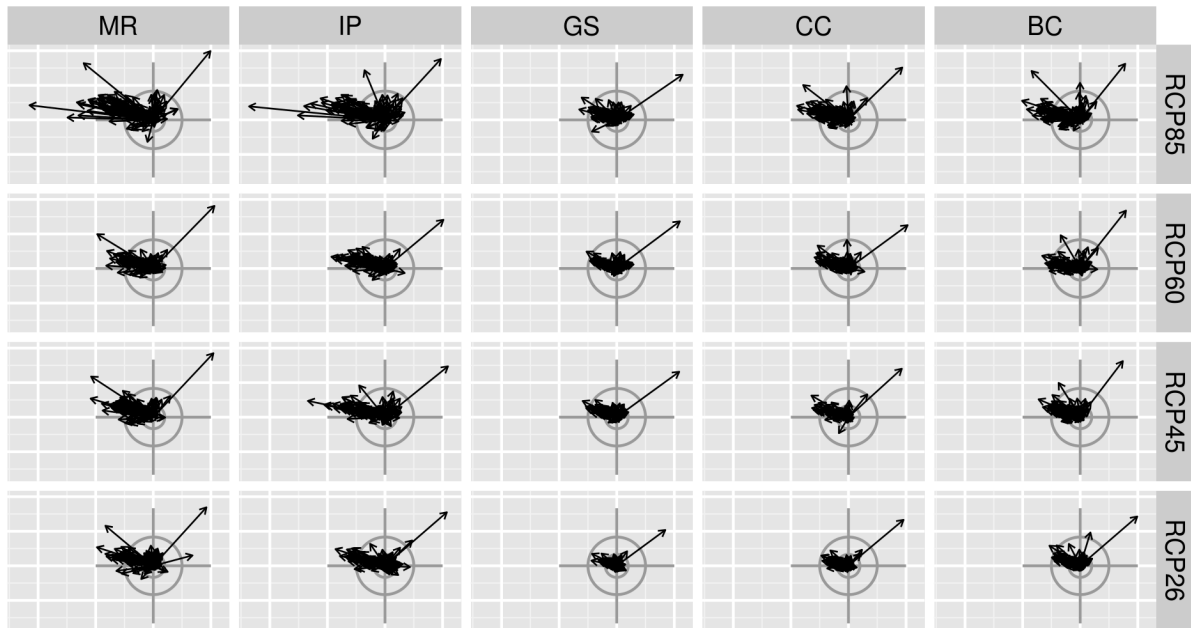

Supplement: Supplemental Information 3 — Y axis presents different AOGCM models. X axis presents different RCP models. The arrow and wind rose are same as Fig. 1. [file peerj-04-2185-s003.pdf]

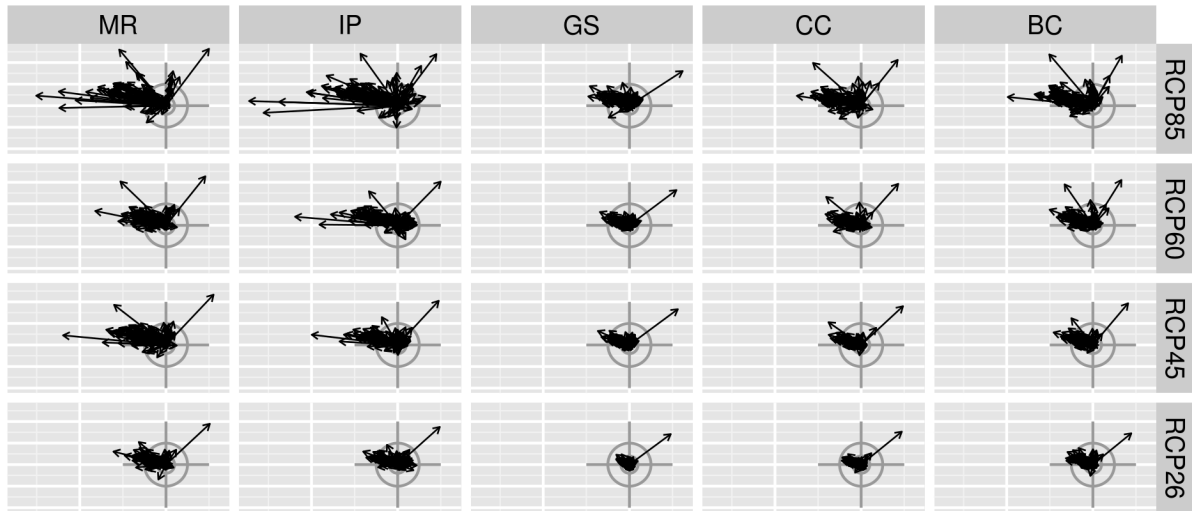

Supplement: Supplemental Information 4 — Y axis presents different AOGCM models. X axis presents different RCP models. The arrow and wind rose are same as Fig. 1. [file peerj-04-2185-s004.pdf]

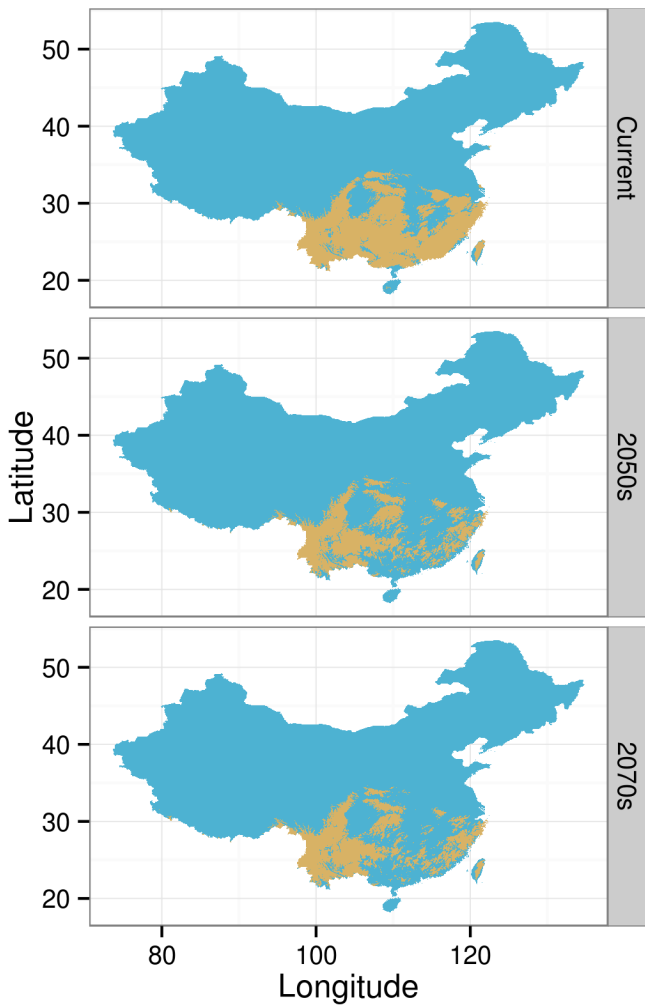

Supplement: Supplemental Information 5 — The figure was generated using R (http://www.R-project.org/), ggplot2 (http://had.co.nz/ggplot2/boo) and raster (http://CRAN.R-project.org/package=raster) softwares, and the maps were created using data downloaded from the GADM database (http://www.gadm.org/) for free use. [file peerj-04-2185-s005.pdf]
